# Investigating the role of *BCAR4* in ovarian physiology and female fertility by genome editing in rabbit

**DOI:** 10.1038/s41598-020-61689-6

**Published:** 2020-03-19

**Authors:** Maud Peyny, Peggy Jarrier-Gaillard, Laurent Boulanger, Nathalie Daniel, Sébastien Lavillatte, Véronique Cadoret, Pascal Papillier, Danielle Monniaux, Nathalie Peynot, Véronique Duranthon, Geneviève Jolivet, Rozenn Dalbies-Tran

**Affiliations:** 1 INRAE, CNRS, IFCE, Université de Tours, PRC, 37380 Nouzilly, France; 2 Université Paris-Saclay, INRAE, ENVA, UVSQ, BREED, 78350 Jouy-en-Josas, France; 3 INRAE, PFIE, 37380 Nouzilly, France; 4 CHU Bretonneau, Médecine et Biologie de la Reproduction-CECOS, 37044 Tours, France

**Keywords:** Functional genomics, Reproductive biology

## Abstract

*Breast Cancer Anti-estrogen Resistance 4* (*BCAR4*) was previously characterised in bovine species as a gene preferentially expressed in oocytes, whose inhibition is detrimental to *in vitro* embryo development. But its role in oogenesis, folliculogenesis and globally fertility *in vivo* remains unknown. Because the gene is not conserved in mice, rabbits were chosen for investigation of *BCAR4* expression and function *in vivo*. *BCAR4* displayed preferential expression in the ovary compared to somatic organs, and within the ovarian follicle in the oocyte compared to somatic cells. The transcript was detected in follicles as early as the preantral stage. Abundance decreased throughout embryo development until the blastocyst stage. A lineage of genome-edited rabbits was produced; *BCAR4* expression was abolished in follicles from homozygous animals. Females of wild-type, heterozygous and homozygous genotypes were examined for ovarian physiology and reproductive parameters. Follicle growth and the number of ovulations in response to hormonal stimulation were not significantly different between genotypes. Following insemination, homozygous females displayed a significantly lower delivery rate than their heterozygous counterparts (22 ± 7% vs 71 ± 11% (mean ± SEM)), while prolificacy was 1.8 ± 0.7 vs 6.0 ± 1.4 kittens per insemination. In conclusion, *BCAR4* is not essential for follicular growth and ovulation but it contributes to optimal fertility in rabbits.

## Introduction

Over the past twenty years, the combination of expressional and functional genomics has unveiled a number of mammalian genes whose preferential expression in the oocyte is often associated with a role in ovarian folliculogenesis, fertilisation, or early embryonic development^1–3^. Transcripts are synthesised throughout oocyte growth; subsequently transcription becomes progressively inactivated as the oocyte reaches its full size within the late antral growing follicle. After ovulation and fertilisation, minimal transcriptional activity can be detected in the early embryo. Major embryo genome activation (EGA) occurs after one (in mice) or several cell divisions, as late as the 8/16-cell stage in bovine and rabbit embryos (reviewed in^4^). It is worth noting that, while the majority of genes are transcribed at this stage, oocyte-specific genes are not re-activated. The corresponding transcripts continue to decrease in abundance, becoming undetectable, as shown by transcriptomic approaches in mouse and bovine^5,6^. This is referred to as a maternal profile, as such transcripts are exclusively of maternal origin.

*Breast Cancer Anti-estrogen Resistance 4 (BCAR4*) was discovered in bovine species as an oocyte-preferred expressed gene^6^. Human *BCAR4* was characterised in parallel as a marker of Tamoxifen resistant breast tumours, hence its name^7^, and has since been found to be expressed in various cancers^8^. *BCAR4* transcripts are detected in fully grown oocytes in humans, cows, pigs, dogs and horses^9^. The bovine transcript follows a maternal profile: it decreases throughout embryo development and is not activated at the major embryonic gene activation. A protein is synthesised during oocyte maturation, becomes abundant in the zygote until the 8-cell stage, persists in the morula before becoming undetectable in blastocysts. Following microinjection of siRNA into *in vitro* matured bovine oocytes, BCAR4-knocked-down embryos displayed a compromised development to the morula stage. *BCAR4* appears therefore involved in embryonic development *in vitro*, but its role during *in vivo* embryo development, as well as its expression and function during folliculogenesis, remains uncharacterised. The objective of the current study is to address these gasps in knowledge.

Rabbit was an enticing model organism. An interesting characteristic of the rabbit pertaining to the putative *BCAR4* maternal-effect is the delay in major EGA after several cell divisions. A second argument for rabbit as model organism relates to ovarian development, which is particularly convenient for studying in offspring. In this species, meiosis is initiated at birth in the female gonad. Primordial follicles assemble two to three weeks post-partum (wpp); they can initiate growth around 4 wpp, and antral follicles are detected by 12 wpp^10,11^. We have therefore characterised *BCAR4* expression in the doe. Afterwards, we have implemented genome editing to produce *BCAR4* knocked-out animals. To our knowledge, this is the first report of genome editing of a gene preferentially expressed in oocytes in a non-murine mammal. Females carrying an altered *BCAR4* gene, in parallel with their control wild-type littermates, were analysed on several parameters related to ovarian physiology and reproduction.

## Results

### Identification of the rabbit *BCAR4* orthologue and ovarian expression

**Figure 1:**
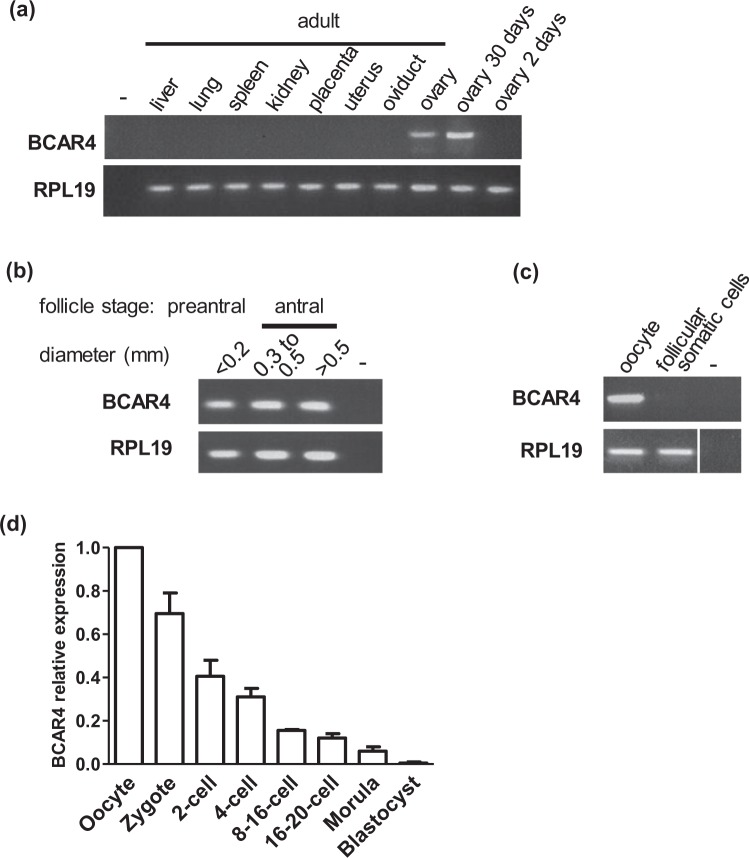
*BCAR4* expression in wild-type rabbit (**a**) in a panel of somatic organs and in the ovary at adulthood, at 1 month old and 2 days old; (**b**) in growing follicles; (**c**) in oocyte and follicle somatic cells (the RPL19 panel is a composite of non-adjacent lanes from a single gel); (**d**) throughout early embryo development (mean ± SEM). (−) indicates the negative control.

A rabbit *BCAR4* orthologue was previously predicted^9^. Based on the rabbit OryCun2.0 genome sequence, it is located onto chromosome Ocu6, with the putative coding region between bases 5,255,378–5,255,713 on the minus strand. Expression was detected by RT-PCR in the adult ovary, but not in the liver, lung, spleen, kidney, placenta, uterus or oviduct (Fig. 1a and Supplemental Fig. S1a). In the ovary, *BCAR4* transcript was undetected at two days of age, but readily detected at one month, i.e. after follicle formation (Fig. 1a and Supplemental Fig. S1a). Expression was observed as early as the preantral follicle stage and persisted throughout folliculogenesis (Fig. 1b and Supplemental Fig. S1b). In the follicle, *BCAR4* transcript was detected in the oocyte and not in somatic cells (Fig. 1c and Supplemental Fig. S1c).

### Expression throughout embryo development

*BCAR4* transcript was detected by RT-qPCR in ovulated oocytes and its abundance decreased throughout embryo development (Fig. 1d), reminiscent of the bovine profile. It became undetectable in the blastocyst stage. Specifically, *BCAR4 de novo* transcription was not observed at the 8/16-cell stage coincident with major activation of the embryonic genome in the rabbit. This maternal profile is typical of many genes preferentially expressed in the oocyte.

### Phenotyping of *BCAR4*-edited females

#### Oocyte gene expression

**Figure 2:**
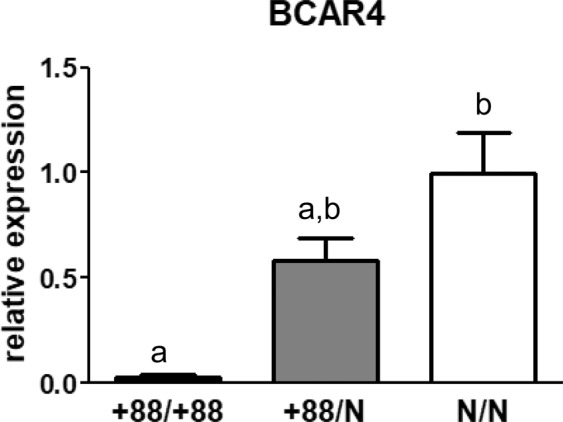
Expression of *BCAR4* in preantral follicles of wild-type and genome-edited animals. Relative transcript abundance, mean ± SEM from analysis of 6 +88/+88 does and 5 +88/N and N/N does. Different superscripts indicate significant differences.

In order to evaluate the role of *BCAR4* in ovarian development and function, a line of rabbits carrying an altered *BCAR4* (labelled +88) was generated (see the Methods section). The impact of the genetic alteration on *BCAR4* expression was analysed by RT-qPCR on preantral follicles with primers targeting a non-edited region of the exon. *BCAR4* transcript was detected at background level in oocytes from all homozygous rabbits, approximately 33-fold lower as compared to wild-type animals (Fig. 2a). This difference was significant (p = 0.027), based on the non-parametric Kruskal-Wallis test followed by post-hoc Dunn’s test (this statistical analysis is used throughout the study, unless otherwise indicated). By contrast, the expression of *Bone Morphogenetic Protein 15 (BMP15)* and *NLR Family Pyrin Domain Containing 5 (NLRP5, also known as MATER*), two oocyte-expressed genes involved in follicle and embryo development respectively, was not significantly affected by the genotype (Supplemental Fig. S2). Altogether, the qPCR data show that *BCAR4* expression is abolished in homozygous animals and that not all oocyte-specific transcripts are affected in *BCAR4* deficient animals.

#### Impact of genome editing onto female fertility

**Figure 3:**
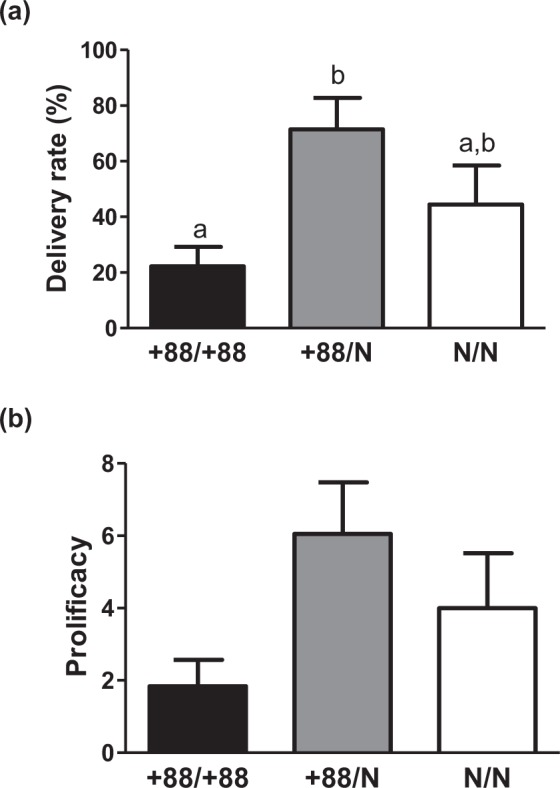
Impact of the genotype onto female fertility. (**a**) Delivery rate and (**b**) prolificacy. (mean ± SEM). Different superscripts indicate significant differences. Analysis from 7 +88/N does and 6 +88/+88 and N/N does.

Wild-type, heterozygous and homozygous females were analysed for changes to reproductive parameters. Following artificial insemination (AI), the number of deliveries, the number and weight of kittens, as well as gestation length were recorded. No abortion was recorded. Parturition started spontaneously, and the genotype did not affect gestation duration (31.5+/−0.3 days, +88/+88; 31.7+/−0.2 days, +88/N; 31.5+/−0.2 days, N/N). The delivery rate per insemination was 22 ± 7%, 71 ± 11% and 44 ± 14% for +88/+88, +88/N and N/N females respectively. It was significantly (p = 0.029) affected by the genotype, lower for homozygous versus heterozygous animals (Fig. 3a). This pattern was repeatedly observed for each of the 3 AI (Supplemental Table S1). Out of three AI, homozygous females had at most one litter, with half of them delivering no viable kittens, while heterozygous and all but one wild-type females had one to three litters (one wild-type female appeared to be infertile). Prolificacy, defined as the mean number of kittens born per insemination, was 1.8, 6.0 and 4.0 for +88/+88, +88/N and N/N genotypes respectively (Fig. 3b). The difference did not reach statistical significance (p = 0.15) due to high inter-individual variability, with ranges of 0–3.7, 1.3–11.3 and 0–10.3 for the three genotypes. Overall, +88/+88 females were subfertile, indicating that the homozygous alteration of *BCAR4* gene is detrimental to female reproduction.

The sex ratio was unaffected by the maternal genotype (Supplemental Fig. S3a) (chi-squared test, number of male and female kittens, p = 0.4). Kittens were weighed within 24 hours of birth. There was also no significant effect of the maternal genotype on average birthweight (59.4 ± 1.4 g, +88/+88; 64.3 ± 1.1 g, +88/N; 63.8 ± 1.1 g, N/N) (Supplemental Fig. S3b).

#### Ovarian activity

**Figure 4:**
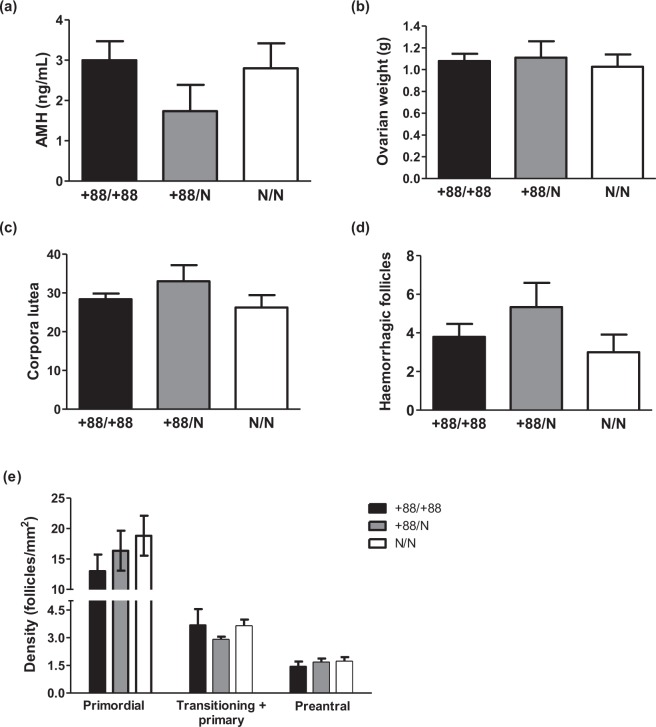
Ovarian physiology. (**a**) AMH concentration in adult female plasma; (**b**) Ovarian weight (sum of left and right ovaries); (**c**) Number of corpora lutea after ovarian stimulation; (**d**) number of haemorrhagic follicles; (**e**) Density of follicles in the ovarian cortex. Mean±SEM from 5 (except e, 6) +88/+88 does, 6 (except b, 4) +88/N does and 4 (except b, 3 and e, 5) N/N does.

As anti-Mullerian hormone (AMH) is secreted by small antral growing follicles, it is a marker used to estimate ovarian activity in various species. As age is known to impact the concentration of AMH in blood as a reflection of decreasing ovarian reserve, attention was paid to the age of animals at blood collection. Mean age was very similar among the three genotypes (within 321–333 days). Concentration of AMH in plasma was within the 1.9–4.6 ng/mL range for wild-type and homozygous carrier animals, and within the 0.9–5.0 ng/mL range for heterozygous animals. No impact of the genotype could be observed, as mean AMH concentrations were 3.0 ± 0.5 ng/mL, 1.7 ± 0.7 ng/mL, and 2.8 ± 0.8 ng/mL for homozygous carriers, heterozygous carriers and wild type animals (Fig. 4a).

Following ovarian stimulation, the animals were sacrificed. Autopsy revealed no obvious anomaly, except for one homozygous carrier (see below). Ovarian weight was not significantly different between genotypes (Fig. 4b). The number of corpora lutea in response to stimulation ranged from 18 to 44. It was not significantly different between genotypes (28.4 ± 1.4, +88/+88; 33.0 ± 4.2, +88/N; 26.3 ± 3.2, N/N) (Fig. 4c). The number of haemorrhagic non-ovulated follicles was also not statistically different (3.8 ± 0.7, +88/+88; 5.3 ± 1.3, +88/N; 3.0 ± 0.9, N/N) (Fig. 4d). Histology of the ovaries showed the presence of follicles from primordial to antral stages for all three genotypes (Supplemental Fig. S4). Numerous primordial follicles with flattened granulosa cells remained in the ovarian reserve. Transitioning follicles had activated and displayed both flattened and cuboidal granulosa cells. Primary and preantral follicles with one or multiple layers of cuboidal granulosa cells respectively were visible. Follicles in each category were counted, and their density in the ovarian cortex was evaluated (Fig. 4e). The differences between the genotypes were not statistically significant. The mean density of primordial follicles was approximately a third lower in ovaries from +88/+88 does compared to wild-type animals, although we did not observe increased atresia at this early stage. The mean density of activated, growing follicles was similar between the two genotypes, with limited inter-individual variability, suggesting that follicle growth is not altered in homozygous animals. Finally, the few healthy antral follicles (Supplemental Fig. S4) were not quantified, as their number may not reflect regular ovarian dynamics due to stimulation with FSH.

Intriguingly, one homozygous carrier had a unique right uterine horn and a shrunken left oviduct (Supplemental Fig. S5). Its right and left ovaries were respectively the lightest and the heaviest (0.37 g and 0.85 g) in the whole group. This long left ovary was devoid of any large follicles on one side, but displayed recent corpora lutea on the other side, and oocytes were recovered by flushing the shrunken oviduct. This female had delivered one litter of two stillborn kittens.

## Discussion

This study shows for the first time that *BCAR4* is transcribed in ovarian preantral follicles. In rabbits, similar to what is observed in cows and humans, the transcript is detected in mature oocytes and persists in early embryos but is not reactivated at the maternal to embryo transition. Females with a non-functional *BCAR4* gene displayed a reduced fertility. We will discuss this phenotype and whether the molecular mechanisms reported to underlie the role of *BCAR4* in cancers may be relevant to its role in reproduction.

Considering genes preferentially expressed in the oocyte, a first class of transcripts and proteins coordinate the development of the oocyte itself and its surrounding follicle. In the current study, we analysed a number of parameters related to this process in wild-type and *BCAR4*-edited does. The density of primordial, transitioning/primary, and preantral follicles in the ovarian cortex was not affected by the genotype, demonstrating that *BCAR4* is not essential for follicle formation or basal growth. This conclusion is consistent with the absence of significant effects of the genotype onto expression of two other oocyte-specific transcripts (BMP15 and NLRP5) in preantral follicles. It can be hypothesised that abnormal oocyte growth would have strongly impacted the oocyte transcriptome; a transcriptomic analysis of oocytes from females of the three genotypes might rather have shown the opposite. Plasma concentration of AMH was within the 0.9–5.0 ng/mL range, consistent with a previous report^12^, and values for wild-type and homozygous does fell within the same range (1,9-4,6 ng/mL). The high inter-individual variability was not unexpected, as it was previously observed in other species including humans, sheep and cows. Both the number of haemorrhagic non-ovulated follicles and the number of corpora lutea were similar to those previously reported in response to stimulation with FSH^13^, and none were significantly altered in *BCAR4*-edited rabbits. These results show that, at least when exogenous FSH is administered, terminal follicular growth does not require *BCAR4* expression. Overall, our study strongly supports the hypothesis that *BCAR4* is dispensable and likely not involved in the whole process of folliculogenesis and ovulation.

A second class of oocyte-expressed genes produce transcripts and proteins that are not involved in oogenesis and folliculogenesis. These products are stored and are recruited following fertilisation to support the development of the transcriptionally quiescent early embryo; they are called maternal-effect genes (reviewed in reference^3^). In our previous work, *BCAR4*-targeting siRNA affected bovine embryo development *in vitro*^9^, supporting the hypothesis that *BCAR4* is a maternal-effect gene. Here, the delivery rate of homozygous +88/+88 females was very low (22 ± 7%), while the AI success rate of heterozygous +88/N females (71 ± 11%) was in the normal range. This low AI success rate in homozygous does, despite a normal folliculogenesis and ovulation, is compatible with a role for *BCAR4* in fertilisation or the early stages of embryo development. These females were not sterile, however, suggesting that *BCAR4* is not absolutely required for these processes *in vivo*. An alternative, compensatory albeit suboptimal mechanism may have been activated in animals with a non-functional *BCAR4* gene. As for the biological process underlying BCAR4 action, bovine embryo development rate was previously observed to be reduced when BCAR4-targeting siRNA were micro-injected into transcriptionally silent mature oocytes^9^, indicating that the effect is likely independent from transcription. It may rather result from post-transcriptional regulation of other maternal-effect transcripts or from stabilising or activating maternal-effect proteins.

The molecular mechanisms underlying the role of *BCAR4* in reproduction have not been thoroughly investigated. But *BCAR4* is also expressed in cancers, and several mechanisms have been proposed to mediate its oncogenic properties. BCAR4 may be able to modulate the ERBB, mechanistic Target Of Rapamycin (mTor), Hedgehog, or Wnt pathways depending on the cellular context. In the following we will consider whether our observations support similar signalling functions for *BCAR4* in the oocyte and early embryo.

In breast cancer derived IPH-926 and MDA-MB-453 cell lines, *BCAR4* encodes a protein, whose pro-proliferative function is mediated by ERBB2 and ERBB3 receptors^14–16^. ERBB2 and ERBB3 may be involved in rodent primordial follicle formation and activation respectively^17,18^, but most likely via somatic cells. If so, *BCAR4* is irrelevant, consistent with normal folliculogenesis in our genetically modified does. Functional contribution of ERBB2 and ERBB3 to early embryo development is not documented, therefore whether *BCAR4*-induced activation of the receptors is important at this stage cannot be addressed. *BCAR4* was also reported to promote chondrosarcoma cell proliferation through activation of mTOR^19^, a downstream target of ERBB2/ERBB3 signalling. Some features of the phenotype of *BCAR4*-deficient rabbits are reminiscent of the impact of oocyte-targeted mTor knock-out (KO) in mouse^20^. Abrogating mTor expression from the primary follicle stage onwards did not affect follicular development up to the preovulatory stage. It did not suppress ovulation, but oocyte nuclear maturation and embryo cleavage and further development were severely compromised. This confirms independent studies reporting that activation of the mTOR-EIF4F pathway is necessary *in vitro* for spindle formation in mouse^21^ and meiotic progression beyond metaphase I in cow^22^, and for cleavage of fertilised mouse eggs^23^. However, if mTOR activation in pronuclear zygotes is conserved in other mammalian species, BCAR4 is likely not essential in the process: otherwise, knocking-down *BCAR4* in mature bovine oocytes would have impacted the first cleavage^9^.

*BCAR4* is also described as a long noncoding RNA partner of the Hedgehog effector GLI2 within a ribonucleoprotein complex that activates a non-canonical Hedgehog/GLI2 transcriptional program^24,25^. Supplementing pig embryo culture medium with Sonic Hedgehog did not impact cleavage but stimulated blastocyst formation^26^. This is opposite to the effect of BCAR4-targeting siRNA in bovine embryos^9^. Thus, at first glance, Hedgehog-induced promotion of embryo development might involve BCAR4. However, considering that a transcriptional response is not expected in pre-EGA embryos, this mechanism is not likely. Interestingly, *Gli2* expression was affected in oocytes from mice with an oocyte-targeted KO of Basonuclin, which share similarities with *BCAR4*-edited rabbits. Basonuclin-depletion had little effect onto folliculogenesis and ovulation rate, but those mice displayed a reduced fertility of variable severity, associated with a low embryo development rate^27^.

A third mechanism was reported in colon cancer: BCAR4 transcript can bind to and stabilize beta-catenin to activate canonical Wnt/beta-catenin signalling^28^. It involves beta-catenin translocation to the nucleus to regulate gene expression. Mice with constitutive activation of this pathway in oocytes exhibited normal folliculogenesis, ovulation and early embryonic development^29^. Yet, such transcription-dependent mechanism is unlikely in transcriptionally quiet bovine or rabbit embryos. In addition to beta-catenin, Wnt can stabilize other proteins involved in cell division^30^. Interestingly, Wnt/stabilization of proteins (Wnt/STOP) signalling functions in metaphase II Xenopus oocytes and is necessary for transcription-independent early cleavage of embryos^31^. It has not been reported in mammals, and this would definitely deserve investigation. In both pathways, Wnt prevents Glycogen Synthase Kinase-3 (GSK3)-mediated phosphorylation of beta-catenin or other proteins, and protects them from subsequent ubiquitination and degradation. Reports on the role of GSK3 in oocytes *in vitro* and *in vivo* appear contradictory. In bovine, GSK3B is progressively inactivated over the course of *in vitro* maturation, and metaphase II oocytes contain inactive GSK3B^32^ as do mouse ovulated oocytes^33^. Inhibiting GSK3 during *in vitro* development induced a two-cell block of mouse embryos coincident with major genome activation^33^. This effect is compatible with the efficient cleavage but later developmental arrest of *BCAR4*-knocked-down bovine embryos. *In vivo*, surprisingly, depletion of GSK3 in oocyte from the primary follicle onwards in transgenic mice did not perturb folliculogenesis, ovulation, fertilisation or embryo development^34^. This may indicate a redundant or alternative process *in vivo*, as suggested earlier for *BCAR4*.

In conclusion, we report here the first KO of a maternally expressed gene in a non-murine mammal. Through genome editing in rabbits, we have demonstrated that *BCAR4* is dispensable *in vivo* for folliculogenesis and ovarian activity, but is required for optimal fertility, as homozygous females were subfertile. Based on our results and mechanisms reported in cancer, we can speculate that following ovulation or fertilisation, maternal BCAR4 activates a transcription-independent pathway that contributes to translation or stabilization of maternal proteins involved in sustaining development between the first cleavage and major embryo genome activation.

## Methods

### Animal care

The HY07 line of New Zealand white rabbits (Hypharm, Sèvremoine, France) was used to study *BCAR4* expression in wild-type animals, as well as to generate the genome-edited line (see below). All experimental procedures were approved by CEEA Comethea or Val de Loire ethics committees and by the French Ministry for Education and Research (agreement 13-008, reference APAFIS#1107-2015091713319620v3 and 12072-2017110808307267). Rabbits care and handling were carried in accordance with European regulations on animal welfare.

Ovarian stimulation was induced by five subcutaneous administrations of 5–10 µg FSH (Stimufol®, Reprobiol, Ouffet, Belgium) at 12 hrs intervals. For embryo production, this was followed 6 hrs later by an intravenous administration of 30 IU hCG at the time of natural mating. Alternatively, for phenotyping onto ovarian response to stimulation, 1.6 µg busereline acetate was injected intramuscularly at the time of AI with commercial fresh semen from HY07 rabbits (Hypharm), 2.5 days before slaughtering; the ovaries were weighed and the numbers of recent corpora lutea and non-ovulated haemorrhagic follicles were recorded.

For analysing fertility, a week-long light treatment preceded an injection of 0.8 µg busereline acetate at the time of AI. The protocol was repeated three times six weeks apart for each female.

### Collection of follicles, oocytes and embryos

Preantral and antral follicles were obtained by enzymatic digestion of ovarian strips as described in ewes^35^. Oocyte-cumulus complexes were obtained by aspiration from follicles larger than 0.5 mm in diameter, oocytes were denuded from cumulus cells by repeated pipetting, and aspirated somatic cells were collected.

Ovulated oocytes were recovered at 16 hrs following ovarian stimulation and ovulation induction by flushing the oviducts with phosphate-buffered saline (PBS) containing 10% fetal bovine serum (FBS). Embryos were collected by flushing the genital tract at various stages: one-cell (19 hours postcoitum (hpc)), 2-cell (24 hpc), 4-cell (32 hpc), 8/16-cell (39 hpc), 16/20-cell (56 hpc), morula (67 hpc) and blastocyst (96 hpc). *In vitro* developed embryos at the same stages were obtained by incubating one-cell collected embryos in 500 μL of TCM199 medium (Sigma, Saint-Quentin Fallavier, France) supplemented with 10% FBS at 38 °C under 5% CO_2_ in air for 8, 14, 30, 38, 53 and 79 hrs respectively.

### RNA purification and RT-PCR

Using Trizol (Thermofisher Scientific, Illkirch-Graffenstaden, France), RNA was purified from biopsies of adult liver, spleen, kidney, lung, oviduct, placenta, uterus and ovary, as well as 2-day old and 1-month-old rabbit ovary. 1 µg RNA was treated with DNAse (Promega, Charbonnières-Les-Bains, France). 500 ng was reverse-transcribed with Moloney Murine Leukemia Virus Reverse Transcriptase (Promega) using oligo(dT)15 primers (Promega) in 20 µL final volume. The other 500 ng was used in a mock- reaction without enzyme, as a negative control. 1 µL of reverse-transcription products was used in a 20 µL PCR reaction with 10 µL iQ Sybrgreen Supermix (Biorad, Marne la Coquette, France) and 300 nM gene-specific primers RPL19-F1/R1 or BCAR4-F1/R1 encompassing the putative coding sequence (all primers were from Sigma and their sequences are provided in Supplemental Table S2). The fragments were analysed by electrophoretic migration onto a 1.5% agarose gel containing gelRed (VWR, Fontenay-sous-Bois) and sequenced.

Pools of 15 follicles from individual does were supplemented with 0.1 pg/follicle luciferase RNA (Promega). RNA was purified using the Picopure RNA extraction kit (Thermofisher Scientific) and treated with DNAse (Qiagen, Courtaboeuf, France). Absence of significant DNA contamination was verified by PCR. RNA was submitted to reverse-transcription as above. qPCR was set as above, with gene-specific primers BCAR4-F2/R2 (located within a region conserved in wild-type and genetically altered animals, (Supplemental Fig. S6)), BMP15-F1/R1, NLRP5-F1/R1, luciferase-F1/R1. Dissociation curves displayed a single peak; the amplicons were sequenced to confirm specificity. Efficiency was within the 86–104% range, as calculated based on concomitantly analyzing serial dilutions of a plasmid including the target sequence. Exogenous luciferase was used to normalize expression between samples. Then data were normalised to the expression level in wild-type genotype. The fragments were analysed by electrophoretic migration onto a 1.5% agarose gel. Pools of 15 fully-grown oocytes and corresponding somatic follicular cells underwent a similar protocol for RNA purification and RT, followed by PCR with BCAR4-F2/R2 and RPL19-F1/R1.

For characterizing *BCAR4* transcript profile during embryo development, using Picopure RNA extraction kit as above, DNAse-treated RNA was isolated from batches of at least 20 *in vivo* developed embryos at each stage, or 20 *in vitro* developed embryos at each stage (with 16 *S* and 23 *S* ribosomal RNA as carrier), after supplementation with 1 pg/embryo luciferase RNA. Reverse transcription was performed on 10 ng RNA from *in vivo* developed embryos, or RNA corresponding to 5 *in vitro* cultured embryos at each stage, using Superscript III enzyme (Thermofisher Scientific) and random hexamers (Thermofisher Scientific) in 20 µL final volume. cDNA was diluted 12.5-fold, then 10 µL was used in a triplicate 25 µL PCR reaction with 100 or 200 nM gene specific primers (BCAR4-F2/R2, luciferase-F2/R2) and Sybr Green PCR Master Mix (Thermofisher Scientific). Amplicon specificity was confirmed as above. Efficiencies were calculated based on concomitant analysis of serial dilutions of pre-amplified embryonic cDNA^36^. Data were analysed using QbasePlus software (Biogazelle, Gent, Belgium). Exogenous luciferase was used to normalize expression between samples. Then data were normalised to expression level in ovulated oocytes.

### Ovarian histology

Ovarian biopsies were fixed in Bouin solution (50% saturated picric acid, 3.7% formaldehyde, 5% acetic acid), embedded in paraffin and serially sectioned (7 µm). The ovarian sections were stained with hematoxylin and images acquired with an Axioscan Z1 (Zeiss, Le Pecq, France) slide scanner. The cortex was drawn and its area measured using the Zen software (Zeiss).

### Quantitative analysis of AMH

AMH was measured in 50 µL thawed plasma diluted fourfold with plasma from a castrated male using the AMH GenII ELISA (Beckman-Coulter, Roissy, France) as previously described^12^. Seven twofold serial dilutions of the standard (from 0.078 to 5 ng/mL) diluted in the same plasma from castrated rabbit were analysed in duplicate; the mean of duplicates was used to establish the linear calibration curve (r = 0.999). All experimental values fell within the validated range.

### Generation of rabbits carrying an altered BCAR4

#### Genome editing

The vicinity of the putative translation initiation codon was selected to design a pair of Transcription Activator-Like Effector Nuclease (TALEN) with the ZiFiT Targeter program (http://zifit.partners.org)^37^. A potential TALEN target sequence identified by the program was selected with a preference for an 18-16-18 combination (16 bases for the spacer). The chosen sequences were 5′-TCCCTCTAACCACCGCAG and 5′-ACCCACATTTAACCCTGA for the left and right hemiTALEN respectively (Supplemental Fig. S6). A BLAST analysis onto the whole OryCun2.0 genome did not reveal significant sequence homology with the two hemiTALENs, or with one hemiTALEN in both forward and reverse orientations, at any other location in the rabbit genome that might have represented a likely off-target site.

The TALEN kit used for TALE assembly was a gift from the Keith Joung laboratory (Addgene kit # 1000000017). The TALEN were constructed according to the REAL (Restriction Enzyme And Ligation) assembly method, as described^37^.The left TALEN was constructed by assembling units of the kit in the following order: 7, 12, 17, 25, 27, 15, 16, 21, 27, 12, 16, 22, 27, 12, 17 and 21 (by groups of four units). The entire insert was subcloned into the final JDS74 plasmid opened at the Bsmb1 sites. Similarly, the right TALEN was constructed by assembling units in the following order: 7, 11, 19, 24, 29, 15, 20, 21, 26, 11, 20, 24, 30, 14, 19 and 24. The entire insert was then subcloned into the final JDS78 plasmid. Inserts in both plasmids were sequenced. To prepare RNA for microinjection, 5 μg of each plasmid was linearised with 20 U of Age1 enzyme (New England Biolabs, Evry, France). The linearised fragment was purified by migration on an agarose gel and a Qiaquick gel extraction kit (Qiagen). Messenger RNA was produced from 1 μg of purified linearised plasmid with the ARCA T7 capRNA pol kit (Cellscript, TEBUbio, Le Perray-en-Yvelines, France) and polyadenylated with the polyA polymerase tailing kit (Epicentre, TEBUbio) according to the manufacturer’s instructions. Messenger RNA was purified with a RNeasy minikit (Qiagen), re-suspended in water, then diluted to 100 ng/μl in injection buffer (Millipore, France) and stored at −80 °C until used.

Approximately 5 pL of a solution containing 10 ng/µL of each TALEN in HEPES-buffered TCM199 medium (Sigma) was microinjected into HY07 rabbit one-cell embryos (Hypharm). Injected embryos were washed three times in embryo culture medium TCM199 with 10% FBS. For preliminary *in vitro* validation, the injected embryos were cultured in TCM199 medium containing 10% FBS for 3.5 days until they reach blastocyst stage; 47% alleles carried a mutation, which validated the efficacy of the TALEN pair. For *in vivo* experiments, 15 injected embryos were cultured for 2 hrs before surgical transfer into the oviduct of a recipient doe synchronised by intramuscular injection of 0.5 µg busereline acetate^38^. Six kittens were born and genotyped.

#### Genotyping

**Figure 5:**
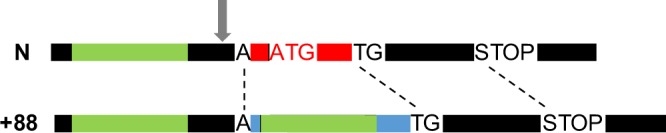
*BCAR4* gene edition. *BCAR4* +88 allele was generated by two insertions of 2 and 16 nucleotides (in blue), a deletion of 63 nucleotides including the first 19 putative codons (in red), and insertion of a fragment of 133 nucleotides (in green) duplicated from an upstream genomic region. The arrow indicates the intron-exon junction.

DNA was extracted from small ear biopsies using the DNeasy Blood and Tissue Kit (Qiagen). Fragments encompassing the expected genomic alteration were amplified using primers BCAR4-F3/R3 and the Kapa2G robust DNA polymerase (Clinisciences, Nanterre, France). They were analysed by electrophoretic migration onto a 1.5% agarose gel, and sent for sequencing with primer R3. The fragment amplified from the wild-type allele was 1178 bp long. Five out of six kittens displayed a genomic alteration. In particular, one female carried a heterozygous deletion of a 63-nucleotide long fragment encompassing the putative Kozak sequence and first 19 codons, as well as insertions of 16 and 2 nucleotides separated by a 133-nucleotide long fragment duplicated from an upstream sequence (Fig. 5 and Supplemental Fig. S6). Overall this resulted into an 88-nucleotide longer allele (+88). This female was selected as the founder of the experimental strain.

#### Generation of experimental animals

After crossing with a male littermate, her first litter included two heterozygous (+88/N) males (as verified by sequencing) which were used as breeders to produce the experimental animals. Their offspring were genotyped by PCR as above: PCR produced a shorter 1178 bp long fragment for wild-type animals (N/N), a longer 1266 bp long fragment for homozygous animals (+88/+88) and both fragments for heterozygous animals (Supplemental Fig. S7). Absence of additional mutation in the region was confirmed by sequencing. Overall, 6 wild-type, 7 heterozygous and 6 homozygous edited does from four litters were included in the subsequent study. These animals grew healthy to adulthood, and remained so throughout the protocol, except for one heterozygous female, which had a difficult third parturition and had to be sacrificed the following day.

When crossing heterozygous animals with heterozygous or wild-type mates, the transmission did not deviate from the expected ratio (Supplemental Table S3). Based on two litters born from crossing heterozygous animals which were sacrificed at birth, the average birthweight was very similar and not statistically different between the kittens genotypes (60.5 ± 5.3 g, N/N; 60.3 ± 2.2 g, +88/N; 60.6 ± 2.3 g, +88/+88).

### Statistics

Using the GraphPad Prism 5 software, the non-parametric Kruskal-Wallis test was applied, followed by Dunn’s test, to analyse the effect of the genotype onto various parameters, except for the impact onto the sex ratio of kittens which was evaluated by the Chi-squared test. P value below 0.05 was considered for significant difference.

## Supplementary information

Supplementary Information.
