# Supplementary material for: Investigating the role of BCAR4 in ovarian physiology and female fertility by genome editing in rabbit
Source: Sci Rep. 2020 Mar 19;10:4992. doi: 10.1038/s41598-020-61689-6 (PMC7081282; doi:10.1038/s41598-020-61689-6)
Supplement: Supplementary file 1 — Supplementary Information. [file 41598_2020_61689_MOESM1_ESM.pdf]

Investigating the role of BCAR4 in ovarian physiology and female fertility by genome editing in rabbit

Maud Peyny[1], Peggy Jarrier-Gaillard[1], Laurent Boulanger[2], Nathalie Daniel[2], Sébastien Lavillatte[3], Véronique Cadoret[1,4], Pascal Papillier[1], Danielle Monniaux[1], Nathalie Peynot[2], Véronique Duranthon[2], Geneviève Jolivet[2] and Rozenn Dalbies-Tran[1,*]

[1] INRAE, CNRS, IFCE, Université de Tours, PRC, 37380 Nouzilly, France

[2] Université Paris-Saclay, INRAE, ENVA, UVSQ, BREED, 78350 Jouy-en-Josas, France

[3] INRAE, PFIE, 37380 Nouzilly, France

[4] CHU Bretonneau, Médecine et Biologie de la Reproduction-CECOS, 37044 Tours, France

* *Correspondence.* INRAE, PRC, 37380 Nouzilly, France; e-mail: rozenn.dalbies-tran@inra.fr

Supplemental informations

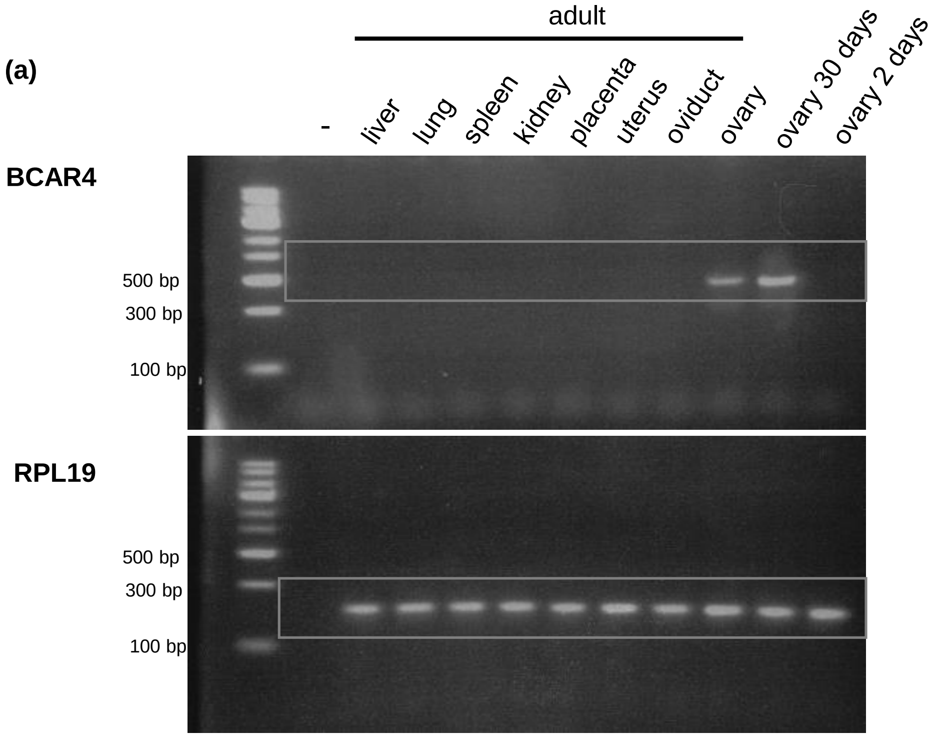

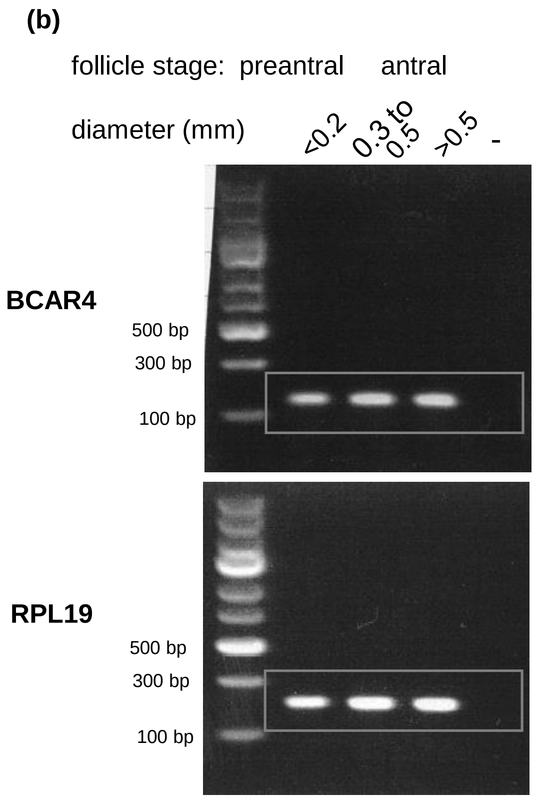

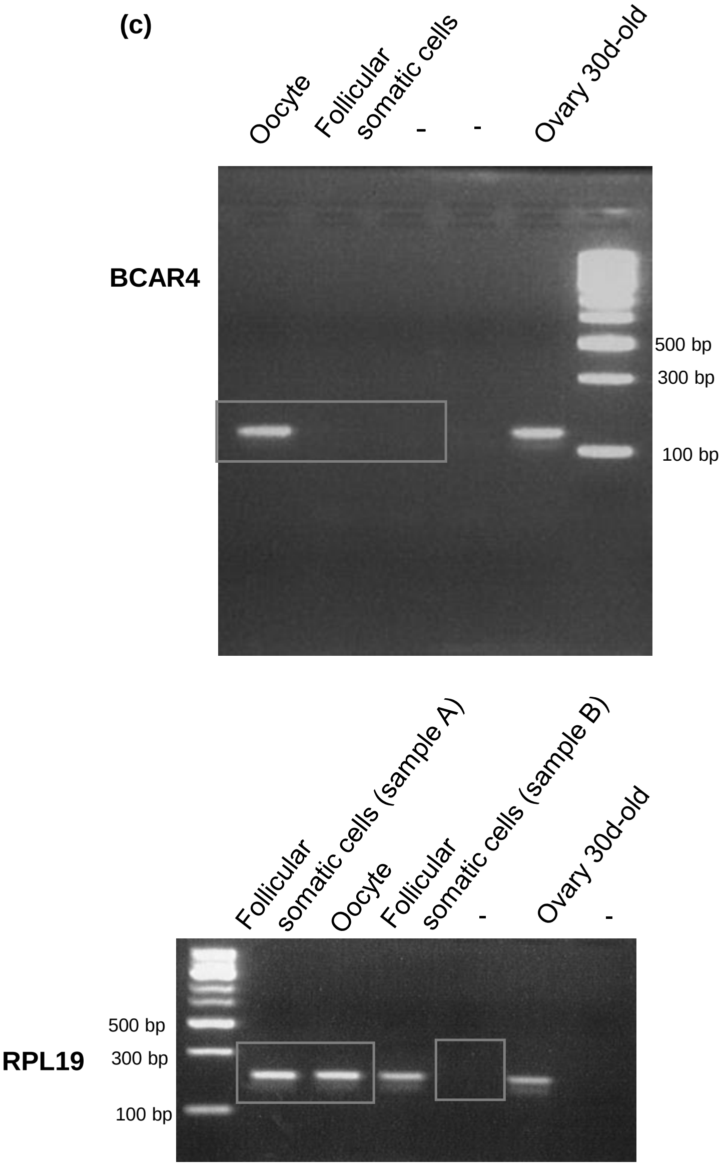

Supplemental Figure S1. *BCAR4* expression in wild-type rabbits (a) in a panel of somatic organs and in the ovary at 1 month old and 2 days old; (b) in growing follicles; (c) in follicular cells. (-) indicates the negative control. Fragments obtained by RT-PCR amplification of regions of RPL19 and BCAR4 (with primers BCAR4-F1/R1 (a) and F2/R2 (b,c)) were separated by electrophoresis in agarose gels. Full-length gels are shown. Boxed regions are displayed in figure 1.

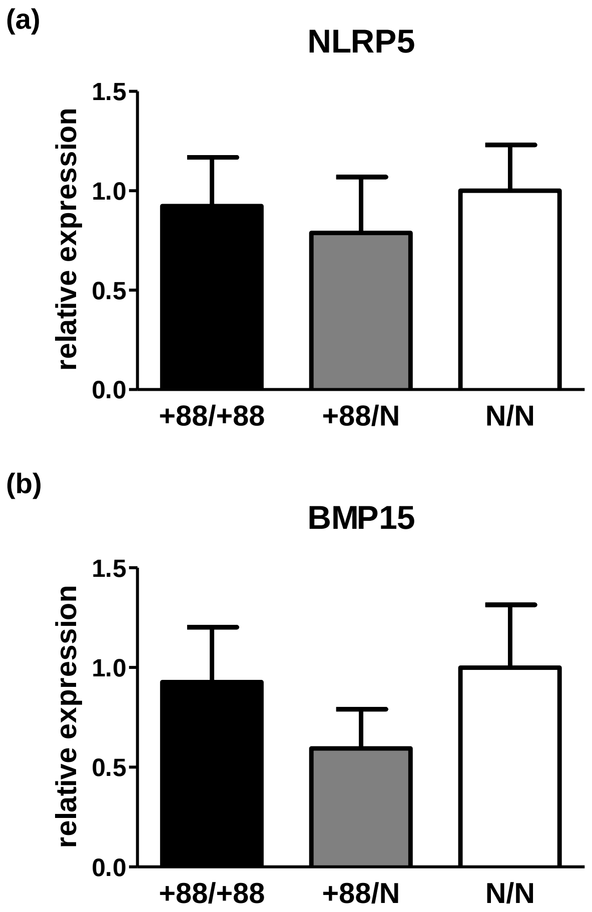

Supplemental Figure S2. Expression of oocyte-preferred genes in preantral follicles of wild-type and genome-edited animals. Relative abundance of NLRP5 (a) and BMP15 (b) transcripts, mean±SEM from analysis of 6 +88/+88 does and 5 +88/N and N/N does.

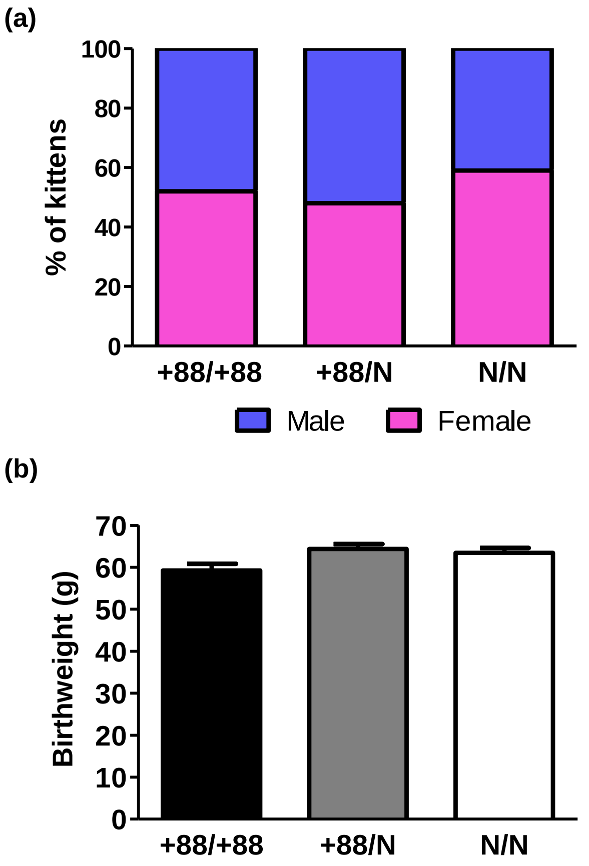

Supplemental Figure S3. Impact of maternal genotype onto offspring. (a) sex ratio; (b) Birthweight (mean ± SEM).

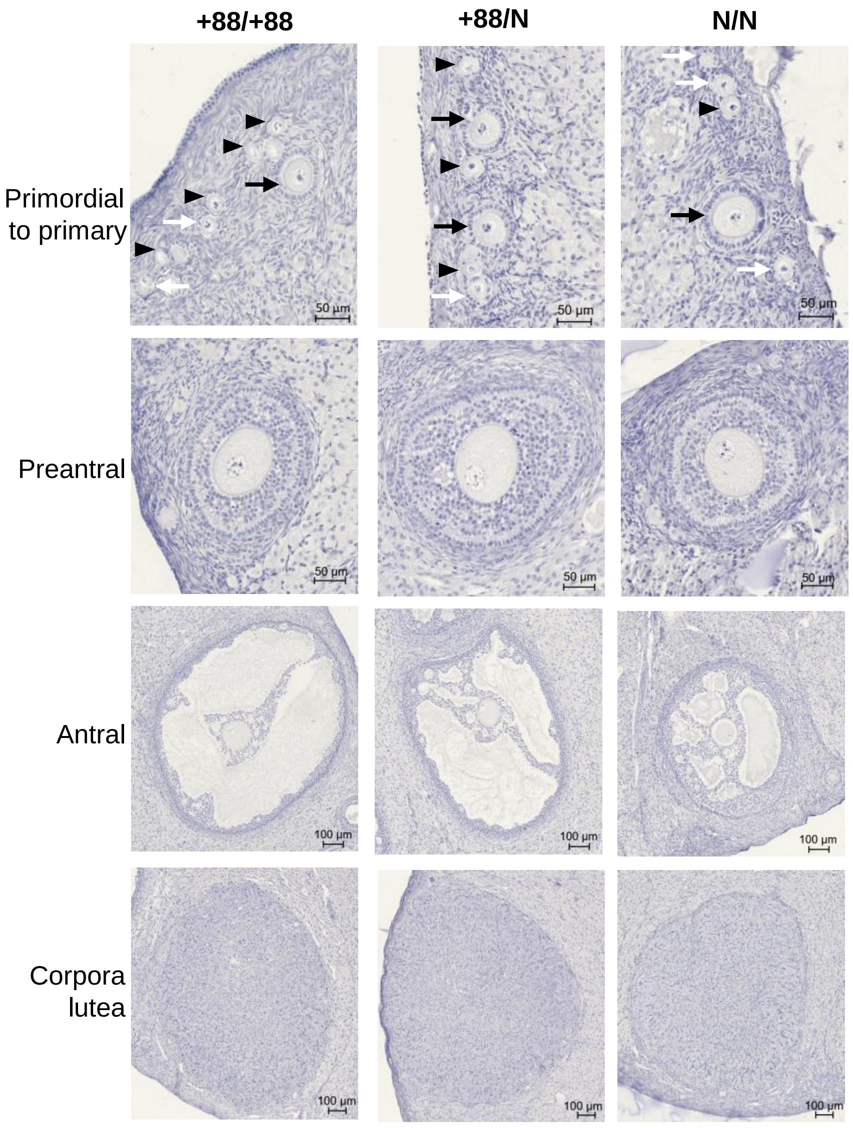

Supplemental Figure S4. Ovarian histology showing follicle development in wild-type and BCAR4-edited does. Representative images of primordial and growing follicles and corpora lutea in ovaries from wild-type (right panel) and genome edited (left and middle panels) does. Arrowheads, white arrows and black arrows show primordial, activated transitioning and primary follicles respectively.

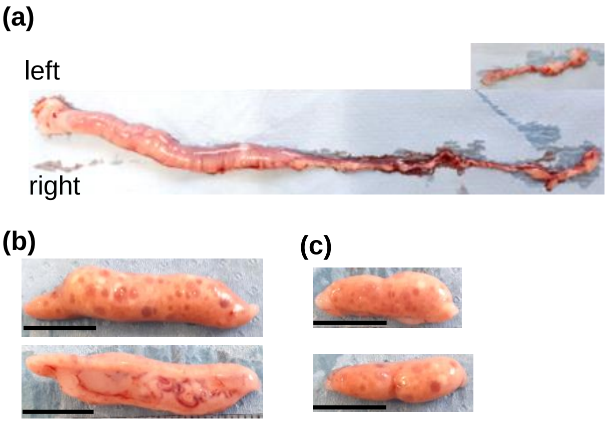

Supplemental Figure S5. Reproductive tract of the one +88/+88 doe displaying anomalies. (a) uterus and oviducts. Both sides of the (b) left ovary and (c) right ovary. Scale bar 1 cm.

```
+88  CCAGGTACATGGATATGGGTTTCACTGAAGGTTTAACTGCTAAGCCAAATGCCAGCGGCATTTTCCCCCAAACTTCGG   Primer F3
N    CCAGGTACATGGATATGGGTTTCACTGAAGGTTTAACTGCTAAGCCAAATGCCAGCGGCATTTTCCCCCAAACTTCGG

+88  AGGGTCCCCATAGCAGCAGCAGGTCCAAGAAGAAAGGTTTCGGCCGGCGCCGCGGCTCACTAGGCTAATCCTCCGCTT
N    AGGGTCCCCATAGCAGCAGCAGGTCCAAGAAGAAAGGTTTCGGCCGGCGCCGCGGCTCACTAGGCTAATCCTCCGCTT

+88  AGCGGCGCCGGCACACCGGGTTCTAGTCCCGGTAGGGGCGCCGGATTCTGTCCCGGTTGCCCTTCTTCCAGGCCAGCC
N    AGCGGCGCCGGCACACCGGGTTCTAGTCCCGGTAGGGGCGCCGGATTCTGTCCCGGTTGCCCTTCTTCCAGGCCAGCC

+88  CTCTGCTGTGGCCAGGGAGTGCAGTGGAGGATGGCCCAGGTGCTTGGGCCCTGCACCCCATGGGAGACCAGGAAAAGC
N    CTCTGCTGTGGCCAGGGAGTGCAGTGGAGGATGGCCCAGGTGCTTGGGCCCTGCACCCCATGGGAGACCAGGAAAAGC

+88  ACCTGGCTCCTGGCTCCTGCCATCGGATCAGCGCGGTGCGCCGGCCGCAGCGCGCCAGCCGCGGCTGCCATTGGAGGG
N    ACCTGGCTCCTGGCTCCTGCCATCGGATCAGCGCGGTGCGCCGGCCGCAGCGCGCCAGCCGCGGCTGCCATTGGAGGG

+88  TGAACCAACGGCAAAGGAAGACCTTTCTCTCTGTCTCTCTCTCTCACTGTCCACTCTGCCTGTCAAAAAAAAAAAAAA
N    TGAACCAACGGCAAAGGAAGACCTTTCTCTCTGTCTCTCTCTCTCACTGTCCACTCTGCCTGTCAAAAAAAAAAAAAA

+88  AAAATACTTGGAGGGGAGAAAGGTTTCTGCTCCACCTTCAGGCTTCGCTCAACCCTGAATTTTTTTTTTCTCCCTCTA
N    AAAATACTTGGAGGGGAGAAAGGTTTCTGCTCCACCTTCAGGCTTCGCTCAACCCTGAATTTTTTTTTTCTCCCTCTA

+88  ACCACCGCAGCACA---------------------------------------------------------------T
N    ACCACCGCAGCACAAGAACACCATGTACCCACATTTAACCCTGAGGGTGCCGAGAAGGCAGGAAGAGCCCGGATGGC-

+88  GTGGGACACCTTCTTGCAGGAAGGCAGGAAGGGGTAAAGACCACGGTCCCGGTAACGGTTCATTGTTGGAAAAGGAAA
N    ------------------------------------------------------------------------------

+88  GGGAAGAACACTCACAGGTGCTGAAAAGACGCAGGACAGAGGGCGTTGTGATCCGCCTCATTGTTGTGCACATGCCTC
N    ------------------------------------------------------------------------TGCCTC

+88  ATTCCGAGTGCCTGCTCGTCACCTGCCTGGGATTGCTGACTGTCACCCTGGGAGCGGTGGTCCTGGGCGGCTCCTCTG   Primer F2
N    ATTCCGAGTGCCTGCTCGTCACCTGCCTGGGATTGCTGACTGTCACCCTGGGAGCGGTGGTCCTGGGCGGCTCCTCTG

+88  ACTTCCTGGCGTTCTCTGCTGGTACAGCAGTGACAGCCGTGGGCGTTTGTTTCGTTGTGTTTTCGGGACTGGTGTTCG   Primer R2
N    ACTTCCTGGCGTTCTCTGCTGGTACAGCAGTGACAGCCGTGGGCGTTTGTTTCGTTGTGTTTTCGGGACTGGTGTTCG

+88  TCTGCAGAGTCAAGCAGAGCCGGCGCAGATGCAGGCTGGAGAGACTCGCAGAGGAAAGAAGGAACGAGAGTGCCGGTG
N    TCTGCAGAGTCAAGCAGAGCCGGCGCAGATGCAGGCTGGAGAGACTCGCAGAGGAAAGAAGGAACGAGAGTGCCGGTG

+88  GCCAGGCCGAGCCCCTGGTGATCGCCAGCACCCGTCTGTAGCCGCCAGCGGAGCCTCGACACTGGCGCACGCGTCGAG   Primer R1
N    GCCAGGCCGAGCCCCTGGTGATCGCCAGCACCCGTCTGTAGCCGCCAGCGGAGCCTCGACACTGGCGCACGCGTCGAG

+88  CTTTTAAATCTTCTGAGCATTGTTTTTTAGATTTAATAAAGTTTGCATTTTAGAAGCCTTTTCTGATCATAGAACTAA
N    CTTTTAAATCTTCTGAGCATTGTTTTTTAGATTTAATAAAGTTTGCATTTTAGAAGCCTTTTCTGATCATAGAACTAA

+88  TTTTTTTTGGAAAAACCTCAAAGCGTGTGTGTGAGAGAGGGAGAAATCGTTGATCGCCTGGTCACTGCTCCCATGCCT
N    TTTTTTTTGGAAAAACCTCAAAGCGTGTGTGTGAGAGAGGGAGAAATCGTTGATCGCCTGGTCACTGCTCCCATGCCT

+88  TCCACAGACAGGACTGGGCCAGGAGCCTGGGTCTCAGTCCAGGTCAGGGGCGGGGACCCAAGTTCTTCAGCCACCACGTTT   Primer R3
N    TCCACAGACAGGACTGGGCCAGGAGCCTGGGTCTCAGTCCAGGTCAGGGGCGGGGACCCAAGTTCTTCAGCCACCACGTTT
```

Supplemental Figure S6. Sequence alignment of the wild-type and edited *BCAR4* loci. *BCAR4* +88 allele displayed two insertions of 2 and 16 nucleotides (in blue), a deletion of 63 nucleotides (in red), and insertion of a fragment of 133 nucleotides (in green) duplicated from an upstream genomic region. The targets of the hemiTALEN are underlined. The putative initiation and stop codon in the wild-type sequence are boldface typed. The arrow indicates the intron-exon junction. The location of primers is in orange (BCAR4-F1 does not appear as it is located in an upstream exon).

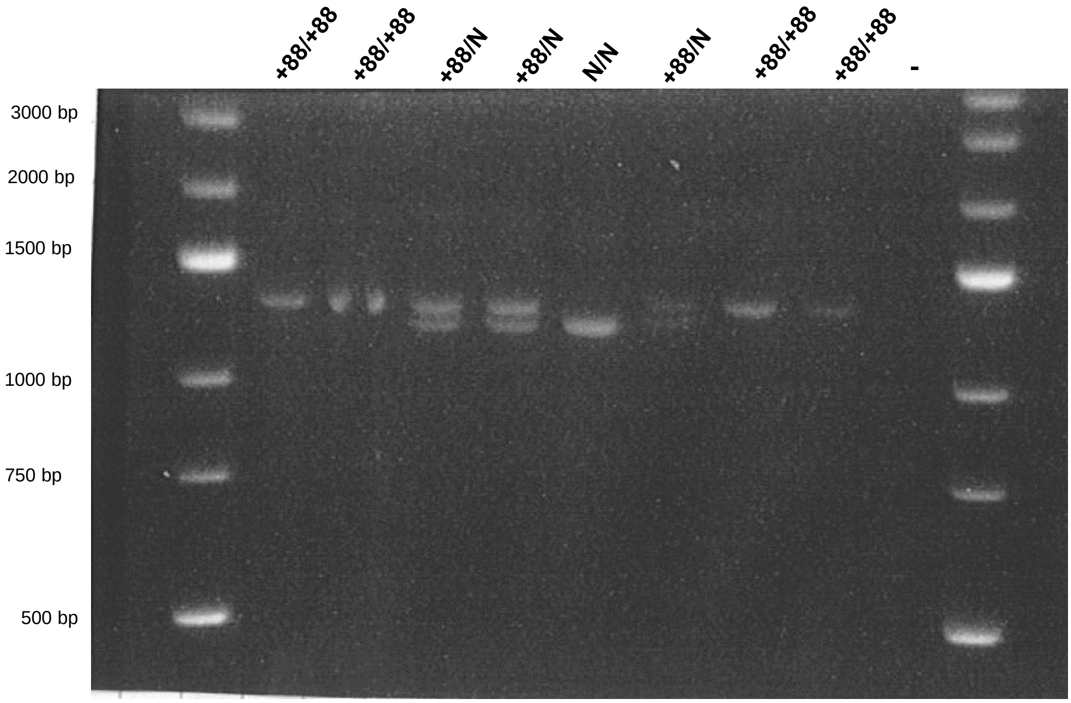

Supplemental Figure S7. Genotyping of rabbits. PCR-amplified genomic fragments from wild-type N/N, heterozygous +88/N and homozygous +88/+88 rabbits analysed by electrophoretic migration in an agarose gel of . (-) indicates the negative control.

Supplemental Table S1. Number of deliveries per

female genotype	number of females	number of deliveries		
		first AI	second AI	third AI
+88/+88	6	1	2	1
+88/N	7	5	4	6
N/N	6	3	2	3

Supplemental Table S2. Primers sequences.

Gene	Forward primer (5'-3')	Reverse primer (5'-3')	Amplicon size (bp)
RPL19	F1 : AATCGCCAATGCCAACTC	R1 : TCATCCAGGTCACCTTCTCC	197
BCAR4	F1 : TGACAGCTTCGCTTTGACTG	R1 : GCTCAGAAGATTTAAAAGCTCGAC	436
	F2 : TCGTCACCTGCCTGGGATT	R2 : CCCGAAAACACAACGAAACAA	130
	F3 : CCAGGTACATGGATATGGGTTTCA	R3 : AAACGTGGTGGCTGAAGAACTTG	wild type : 1178 edited : 1266
BMP15	F1 : ACCATGGTGAGGCTGGTAAG	R1 : GCAGGAGAGACGGAAGTGAG	174
NLRP5	F1 : CAGCACCTGACCAGTCTCAA	R1 : GCCGTCTTCAGTCTTTCCAC	247
Luciferase	F1 : TCATTCTTCGCCAAAAGCACTCTG	R1 : AGCCCATATCCTTGTCGTATCCC	149
	F2 : AGAGATACGCCCTGGTTCCT	R2 : ATAAATAACGCGCCCAACAC	259

Supplemental Table S3. Transmission of the genetic alteration.

Female genotype	Male genotype	Number of kittens		
		N/N	+88/N	+88/+88
N/N	+88/N	23	23	
+88/N	N/N	52	53	
+88/N	+88/N	8	24	13
